# 四钩定位针和记忆合金弹簧圈在肺结节定位中的应用比较

**DOI:** 10.3779/j.issn.1009-3419.2021.102.30

**Published:** 2021-10-20

**Authors:** 星星 薛, 锋 田, 继征 汤, 凯凯 胥, 牧 胡, 永 崔

**Affiliations:** 100050 北京，首都医科大学附属北京友谊医院胸外科 Department of Thoracic Surgery, Beijing Friendship Hospital of Capital Medical University, Beijing 100050, China

**Keywords:** 定位, 胸腔镜, 肺结节, Localization, Thoracoscopy, Pulmonary nodules

## Abstract

**背景与目的:**

随着肺结节微创手术的广泛开展，术前定位变得愈发关键。传统定位方式存在一定的缺陷，定位方式的改进很有必要。本研究旨在对比分析两种新型定位方式即四钩定位针和记忆合金弹簧圈在肺结节定位中应用的安全性和有效性。

**方法:**

回顾性分析152例患者的资料，76例为四钩定位针组，76例为记忆合金弹簧圈组，术前均行肺结节定位，后行电视胸腔镜辅助肺结节楔形切除术，统计平均定位时间、定位并发症、结节切除时间等。

**结果:**

两组患者均成功切除目标肺结节，四钩定位针组76例患者术中均找到定位装置，肺结节全部成功切除，1例因严重胸腔粘连而中转开胸行肺结节楔形切除术。记忆合金弹簧圈组76例患者肺结节均成功切除，1例因术中切除标本后未找到病灶而行妥协性扩大切除术。两组气胸、肺内出血发生率、定位成功率、结节楔形切除时间无统计学差异。四钩定位针组平均定位时间为（13.66±3.11）min，低于记忆合金弹簧圈组的（15.51±3.65）min，二者有统计学差异（*P*=0.001）。记忆合金弹簧圈组当结节至胸膜距离≥1.5 cm和 < 1.5 cm时，平均定位时间分别为（17.20±4.46）min、（14.91±3.15）min，二者有统计学差异（*P*=0.044）。

**结论:**

四钩定位针和记忆合金弹簧圈两种新型肺结节定位方式均有较好的安全性及有效性，四钩定位针定位操作用时更短。在使用记忆合金弹簧圈定位时，对于到胸膜距离 < 1.5 cm的肺结节效果可能更佳。

## Abstract (en)

**Background and objective:**

With the extensive development of minimally invasive surgery for pulmonary nodules, preoperative localization becomes more and more critical. There are some defects in traditional localization methods, so it is necessary to improve. The aim of this study was to compare and analyze the safety and effectiveness of two new methods, namely four-hook needle and memory alloy coil, in the localization of pulmonary nodules.

**Methods:**

A retrospective analysis of 152 patients was performed. 76 cases were in four-hook needle group, and 76 cases were in memory alloy coil group. Pulmonary nodules were located before operation, and then video-assisted wedge resection was performed. The average procedure time, localization complications and nodule resection time were counted.

**Results:**

The target pulmonary nodules were successfully removed in both groups. In four-hook needle group, 76 patients found localization devices, all the pulmonary nodules were successfully removed, and one case was transferred to open the chest for wedge resection of pulmonary nodules due to severe thoracic adhesion. All 76 patients in memory alloy coil group were successfully resected with pulmonary nodules, and one patient underwent compromising enlarged resection because no lesion was found after the specimen was removed during the operation. There was no significant difference in the incidence of pneumothorax and pulmonary hemorrhage, the success rate of localization and nodule wedge resection time between the two groups. The average time of localization in four-hook needle group was (13.66±3.11) min, lower than that of memory alloy coil group (15.51±3.65) min, and the difference was statistically significant (*P*=0.001). In memory alloy coil group, when the distance from the nodule to the pleura was ≥1.5 cm and < 1.5 cm, the average localization time was (17.20±4.46) min and (14.91±3.15) min, respectively, and there was a statistical difference between the two distance (*P*=0.044).

**Conclusion:**

Four-hook needle and memory alloy coil have good safety and effectiveness, and the localization time of four-hook needle is shorter. When using memory alloy coil, the effect of the method is better for pulmonary nodules with a distance less than 1.5 cm to pleura.

对于肺结节的处理目前多采用电视辅助胸腔镜手术（video-assisted thoracoscopic surgery, VATS）行局限性肺切除，根据术中冰冻结果决定术式^[1]^。术前定位愈发成为其中的关键环节^[2]^，可明显影响VATS中转开胸率及手术成功率^[3]^。众多的定位方式中，电子计算机断层扫描（computed tomography, CT）引导下Hook-wire定位和微弹簧圈定位是目前应用最为普遍的定位技术^[4]^。传统Hook-wire定位常见缺点为脱钩、疼痛体验、气胸等并发症以及定位与手术间隔时间较短等，传统微弹簧圈存在移位、需术中再次放射定位等缺陷^[5]^。本中心率先使用两种传统定位方式改良版即四钩定位针和记忆合金弹簧圈同时应用于临床，四钩定位针在传统钩针基础上前端创新性采用金属四钩锚定肺结节，尾端采用标记丝线替代金属丝，记忆合金弹簧圈采用镍钛合金材料制成，在温度 > 30 ℃时可形成独特哑铃状结构锚定于肺结节。本文通过回顾性对比研究，分析探讨两种改良定位方式的临床应用特点及前景。

## 1 资料与方法

### 1.1 一般资料

回顾性分析我院2019年2月-2020年9月收治的152例肺结节患者，术前随机选择定位方式，根据定位方式的不同，分为四钩定位针组（*n*=76）和记忆合金弹簧圈组（*n*=76）。两组患者一般资料见表 1，两组病例特点比较无统计学差异（*P* > 0.05），具有可比性。经医院伦理会批准，患者在定位操作前，均签署知情同意书。纳入标准：①术前胸部CT示肺外结节，结节距离胸膜 < 4.0 cm；②行单个肺结节定位；③符合肺结节手术指征，术者评估触诊困难有定位必要；④术中先行楔形病灶结节切除，根据术中冰冻病理结果决定术式；⑤知情同意。排除标准：①影像学资料不全者；②行多结节定位切除术；③患者病灶位置不适合经皮穿刺定位；④入院时合并气胸、胸腔积液；⑤定位后术中直接行肺段切除或肺叶切除术。

**1:** 两组患者的临床特点 Clinical characteristics of the two groups

Clinical characteristics	Four-hook needle group (*n*=76)	Memory alloy coil group (*n*=76)	*P*
Gender			0.599
Male	25 (31.4%)	22 (28.9%)	
Female	51 (68.6%)	54 (71.1%)	
Age (Mean±SD, yr)	54.55±11.79	58.03±12.75	0.083
Density of nodules			0.261
Pure-solid GGN	30 (39.5%)	40 (52.6%)	
Partly-solid GGN	31 (40.8%)	25 (32.9%)	
Solid nodule	15 (19.7%)	11 (31.4%)	
Diameter of nodules (Mean±SD, mm)	10.44±4.49	10.00±3.33	0.912
Distance from nodule to pleura (Mean±SD, mm)	8.13±7.75	9.34±8.69	0.503
Distance from nodule to pleura			0.243
Distance from nodule to pleura ≥1.5 cm	14 (18.4%)	20 (26.3%)	
Distance from nodule to pleura < 1.5 cm	62 (81.6%)	56 (73.7%)	
Nodules location			0.535
Right upper lobe	25 (32.9%)	17 (22.4%)	
Right middle lobe	3 (3.9%)	4 (5.3%)	
Right low lobe	17 (22.4%)	15 (19.7%)	
Left upper lobe	21 (27.6%)	25 (32.9%)	
Left low lobe	10 (13.2%)	15 (19.7%)	
GGN: ground-glass nodule.			

### 1.2 统计指标

包括定位操作相关指标和定位后手术相关指标。定位操作指标包括定位时间、定位并发症、定位成功率，手术相关指标包括手术成功率、结节楔形切除时间。患者于CT室行定位前第一次扫描至定位结束最后一次扫描视为定位时间，术中根据定位装置而切除的标本中找到病灶视为定位成功，从切皮开始至肺结节楔形切除并找到病灶视为结节楔形切除时间，切除标本中找到病灶视为手术成功。

### 1.3 材料

CT机：Siemens CT机；四钩定位针（一次性使用肺结节定位针）：宁波胜杰康生物科技有限公司；记忆合金弹簧圈：江苏诺瑞思医疗器械有限公司。

### 1.4 定位方法

根据患者术前影像学资料及手术方式选择合适体位，放置定位标志，行CT扫描确定进针位置及进针深度并标记，常规消毒铺无菌洞巾，2%利多卡因局部浸润麻醉。

四钩定位针组：取定位穿刺针，CT引导下将穿刺针穿过胸壁、胸膜，到达肺结节或其边缘，使用推送针释放四钩定位针，将推送针完全拔出穿刺针外，后撤穿刺针，使其针尖位于脏层胸膜外；将推送针再次插入穿刺针，释放尾端定位线；CT确定位置后，将穿刺针、推送针一起拔出体外。

记忆合金弹簧圈组：取穿刺针穿过胸壁至小结节临近肺组织，去掉穿刺针内芯；插入带连接头的导引针，将连接头滑动至穿刺针顶端并旋转固定；根据穿刺距离使用推送针释放头端弹簧圈到合适位置成螺旋状结构；控制连接头，将引导针和穿刺针整体退至推送针退出标记处；将推送针完全推送至抵触导引针，此时弹簧圈尾端完全释放于肺脏层胸膜表面或部分位于胸壁；CT确定位置后，将穿刺针、导引针、推送针整体拔出。

定位操作均由胸外科同一医师完成，两种定位操作示意图及定位装置实物图见图 1、图 2。定位操作及定位后CT表现见图 3。

**1:**
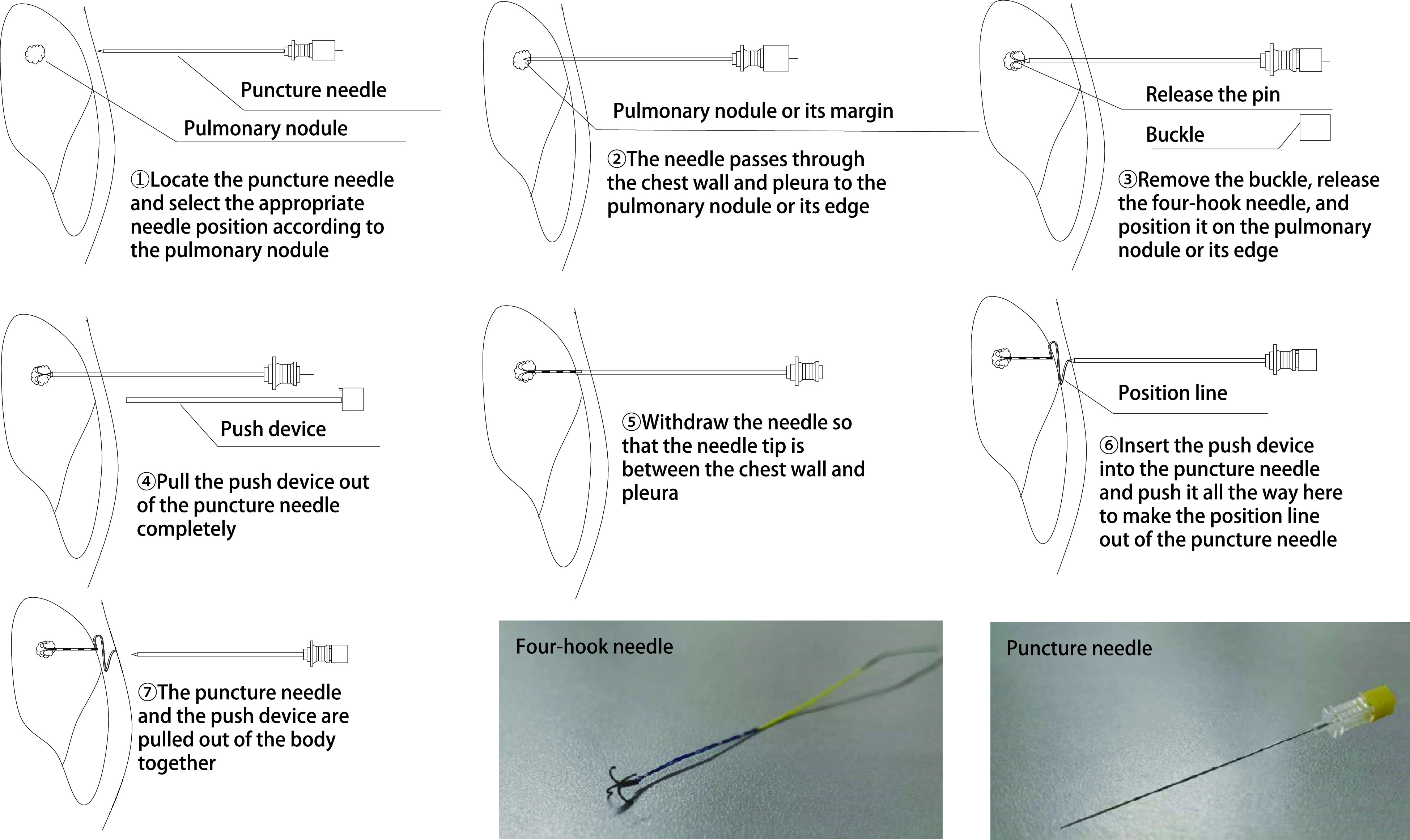
四钩定位针定位操作示意图及定位装置实物图 Operation diagram and physical drawing of four-hook needle

**2:**
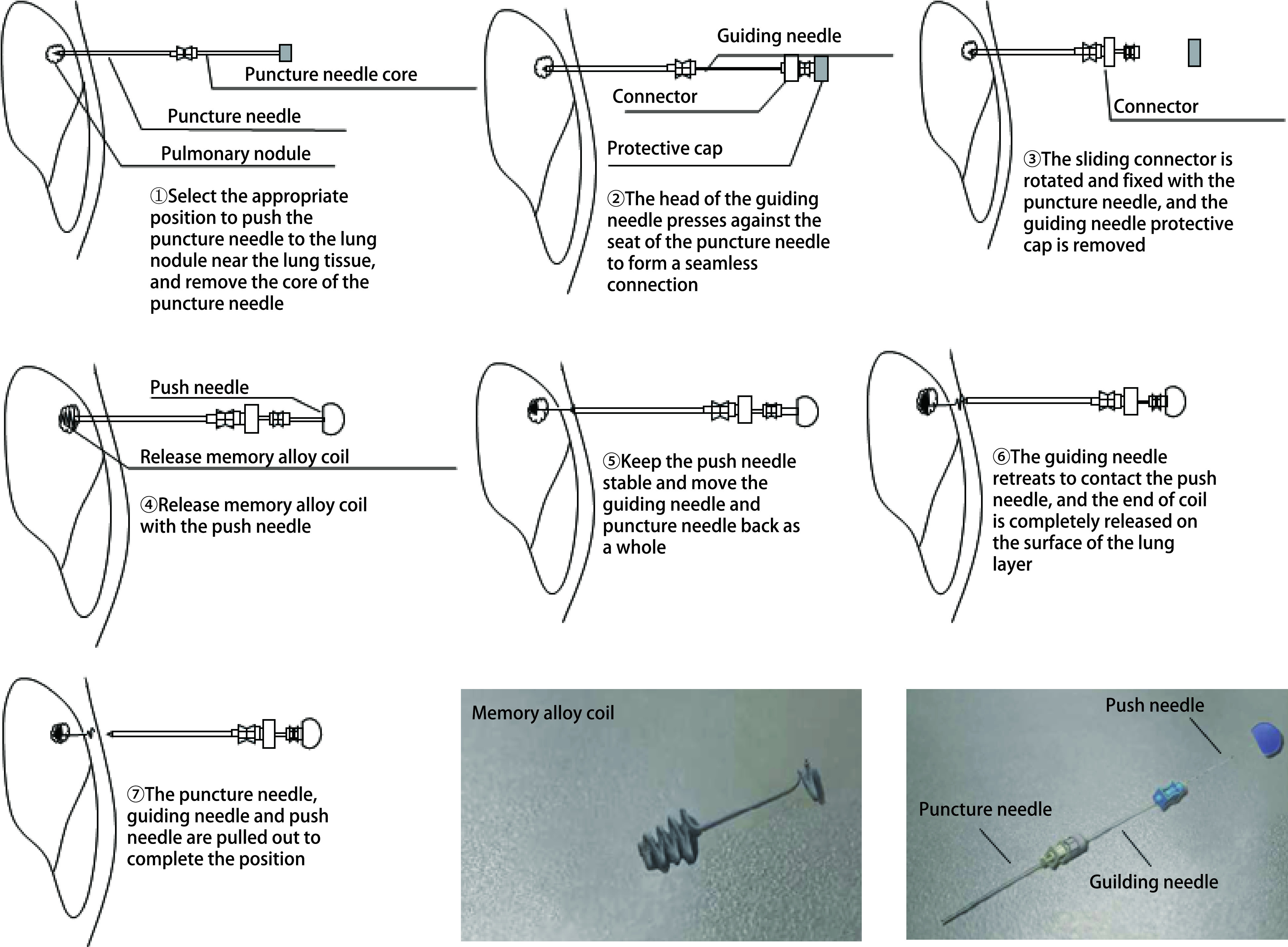
记忆合金弹簧圈定位操作示意图及定位装置实物图 Operation diagram and physical drawing of memory alloy coil

**3:**
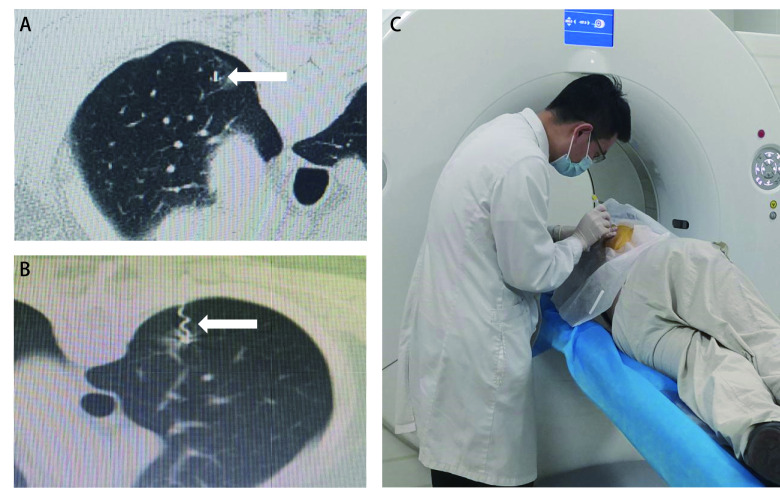
定位操作及定位后CT表现。A：四钩定位针定位后CT表现（箭头）；B：记忆合金弹簧圈定位后CT表现（箭头）；C：定位操作图。 Operation process of positioning and CT performance after localization. A: CT performance after localization of four-hook needle (arrow); B: CT performance after localization of memory alloy coil (arrow); C: Operation diagram.

定位后当日或次日行VATS手术。

### 1.5 手术方法

平卧位全麻气管插管后，行常规VATS手术，进入胸腔后在胸腔镜辅助下寻找定位装置，行肺结节楔形切除，剖开肺组织标本查看定位装置是否完整，并找寻肺结节病灶，若未找到病灶提示定位失败则行扩大切除。两组定位装置术中情况见图 4。术中送快速冰冻，病理结果为早期癌变及良性病变则终止手术，若结果为浸润癌或转移癌，则继续下一步手术。

**4:**
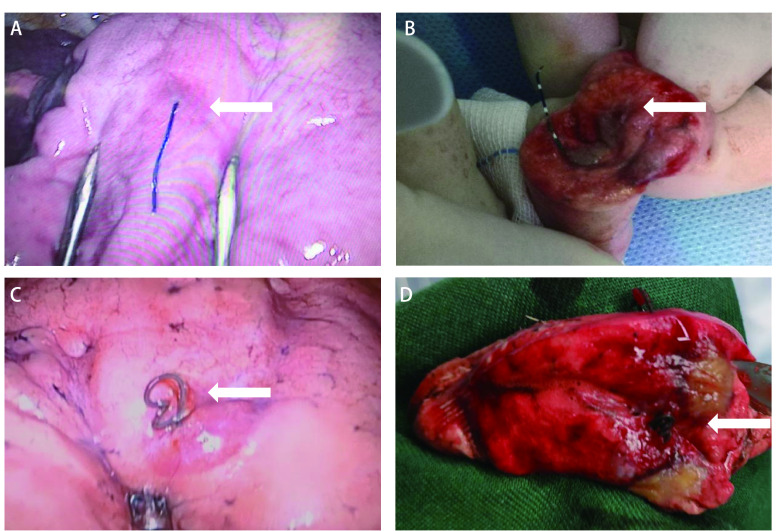
定位装置术中情况。A：四钩定位针定位后术中图（箭头）；B：四钩定位针肺结节标本图（箭头为病灶）；C：记忆合金弹簧圈定位后术中图（箭头）；D：记忆合金弹簧圈肺结节标本图（箭头为病灶）。 Intraoperative situation of localization device. A: Intraoperative picture after localization of four-hook needle (arrow); B: Pulmonary nodule specimen picture after localization of four-hook needle (arrow is lesion); C: Intraoperative picture after localization of memory alloy coil (arrow); D: Pulmonary nodule specimen picture after localization of memory alloy coil (arrow is lesion).

### 1.6 统计学方法

采用SPSS 22.0统计学软件，计数资料采用例数和百分比（%）表示，行卡方检验；计量资料采用均数±标准差（Mean±SD）表示，行*t*检验，*P* < 0.05为差异具有统计学意义。

## 2 结果

### 2.1 定位操作相关结果

四钩定位针组76例（100.0%）患者均成功定位，记忆合金弹簧圈组75例（98.7%）患者成功定位，1例（1.3%）因术中切除标本后未找到病灶而行妥协性扩大切除术，视为定位失败，二者定位成功率无统计学差异（*P* > 0.05）。四钩定位针组发生气胸、肺内出血分别为9例（11.8%）、6例（7.9%），记忆合金弹簧圈组分别为7例（9.2%）、9例（11.8%），两组定位并发症无统计学差异（*P* > 0.05），两组均未出现严重操作并发症，未特殊处理。四钩定位针组平均定位时间为（13.66±3.11）min，记忆合金弹簧圈组为（15.51±3.65）min，二者有统计学差异（*P*=0.001）。在结节距离胸膜≥1.5 cm时，四钩定位针组平均定位时间为（13.86±2.83）min，记忆合金弹簧圈组为（17.20±4.46）min，二者比较有统计学差异（*P*=0.019）；当结节距离胸膜 < 1.5 cm时，四钩定位针组平均定位时间为（13.61±3.19）min，记忆合金弹簧圈组为（14.91±3.15）min，二者比较有统计学差异（*P*=0.028）。四钩定位针组在定位至胸膜不同距离肺结节时平均定位时间无统计学差异，记忆合金弹簧圈组当结节距离胸膜≥1.5 cm时，平均定位时间为（17.20±4.46）min，当结节距离胸膜 < 1.5 cm时，平均定位时间为（14.91±3.15）min，二者比较有统计学差异（*P*=0.044）。定位操作相关结果见表 3。

**2:** 两组定位操作相关结果 Operation related parameters of the two groups

Variables	Four-hook needle group (*n*=76)	Memory alloy coil group (*n*=76)	*P*
Pneumothorax	9 (11.8%)	7 (9.2%)	0.597
Intrapulmonary hemorrhage	6 (7.9%)	9 (11.8%)	0.415
Successful localization	76 (100.0%)	75 (98.7%)	> 0.999
Average procedural time (Mean±SD, min)	13.66±3.11	15.51±3.65	0.001
Distance from nodule to pleura ≥1.5 cm	13.86±2.83	17.20±4.46	0.010
Distance from nodule to pleura < 1.5 cm	13.61±3.19	14.91±3.15	0.028

**3:** 两组定位操作相关结果 Operation related parameters of the two groups

Group	Average procedural time (Mean±SD, min)		*P*
	≥1.5 cm	< 1.5 cm	
Four-hook needle group (*n*=76)	13.86±2.83	13.61±3.19	0.264
Memory alloy coil group (*n*=76)	17.20±4.46	14.91±3.15	0.044

### 2.2 手术相关结果

四钩定位针组76例患者目标结节均成功切除，其中1例因严重胸腔粘连而行中转开胸术，记忆合金弹簧圈组76例结节均成功切除，1例因术中切除标本后未找到病灶而行妥协性扩大切除术。四钩定位针组平均结节切除时间为（22.91±4.45）min，记忆合金弹簧圈组平均结节切除时间为（21.97±4.04）min，两组无统计学差异（*P* > 0.05）。术后病理：四钩定位针组非典型腺瘤样增生3例（3.9%），肉芽肿性结节5例（6.6%），炎性病变8例（10.5%），原位腺癌19例（25.0%），微浸润腺癌22例（28.9%），浸润腺癌13例（17.1%），转移癌3例（3.9%），微小脑膜瘤样结节1例（1.3%），鳞癌1例（1.3%），硬化性肺细胞瘤1例（1.3%）；记忆合金弹簧圈组非典型腺瘤样增生3例（3.9%），肉芽肿性结节1例（1.3%），炎性病变13例（17.1%），错构瘤1例（1.3%），原位腺癌25例（32.9%），微浸润腺癌22例（28.9%），浸润腺癌7例（9.2%），转移癌3例（3.9%），类癌1例（1.3%），手术相关结果见表 4。

**4:** 两组手术相关结果 Surgery related parameters of the two groups

Variables	Four-hook needle group (*n*=76)	Memory alloy coil group (*n*=76)	*P*
Nodule resection time (Mean±SD, min)	22.91±4.45*	21.97±4.04*	0.181
Granulomatous lesions			
Atypical adenomatous hyperplasia (AAH)	3 (3.9%)	3 (3.9%)	-
Postoperative pathology	5 (6.6%)	1 (1.3%)	-
Inflammatory lesions	8 (10.5%)	13 (17.1%)	-
Adenocarcinoma *in situ* (AIS)	19(25.0%)	25 (32.9%)	-
Minimally invasive adenocarcinoma (MIA)	22 (28.9%)	22 (28.9%)	-
Invasive adenocarcinoma	13 (17.1%)	7 (9.2%)	-
Metastatic tumor	3 (3.9%)	3 (3.9%)	-
Hamartoma	0	1 (1.3%)	-
Microneuromatous nodule	1 (1.3%)	0	-
Squamous carcinoma	1 (1.3%)	0	-
Sclerosing pulmonary cell tumor	1 (1.3%)	0	-
Carcinoid	0	1 (1.3%)	-
*: There was no statistical analysis of the patients with positioning failure and conversion to thoracotomy.			

## 3 讨论

随着对于肺结节的不断研究，亚肺叶切除已经成为了推荐的术式^[6, 7]^。对于部分位于胸膜下的肺小结节，术中常常难以通过肉眼及触摸来确定结节位置，术前对于肺结节的精准定位也就变得愈发关键，目前在临床应用的肺结节定位方法众多，而Hook-wire、微弹簧圈临床应用最为广泛^[2]^。传统Hook-wire缺陷在于易出现移位，发生气胸、肺内出血等并发症，定位后疼痛感较强，并需要短时间内进行手术^[8]^，甚至需要杂交手术室^[9, 10]^。本研究中采用的四钩定位针，其头端的金属四钩可减少移位脱钩情况的发生，本研究中未出现脱钩情况，良好的锚定效果延长了定位后到术前的间隔时间，使杂交手术室不再必要。尾端的标记线性结构，有效减轻了患者定位后胸膜移动产生的疼痛体验，线性标记让术者更容易术中判断结节位置。传统微弹簧圈缺陷在于脱位，需术中重复CT扫描，也有因定位失败而导致手术失败的报道^[11]^。本研究所使用的记忆合金弹簧圈采用新型镍钛合金材质，独特的留尾结构更好地方便术者术中寻找定位标志，且无需术中CT再次确认位置，本研究中仅出现1例疑似移位，哑铃状定位结构，降低了移位发生率^[12]^。

在本研究两种新型定位方式对比分析中，四钩定位针组76例（100.0%）患者均成功定位，记忆合金弹簧圈组75例（98.7%）患者成功定位，1例（1.3%）定位失败，二者定位成功率无统计学差异，与文献^[5, 13]^报道的定位成功率相近并稍占优势。两组目标结节均成功切除，四钩定位针组1例因严重胸腔粘连行中转开胸术，记忆合金弹簧圈组1例因术中切除标本后未找到病灶而行妥协性扩大切除术。四钩定位针组出现气胸9例（11.8%），肺内出血6例（7.9%）；记忆金弹簧圈组分别为7例（9.2%）、9例（11.8%），两组出现的并发症均较轻，未特殊处理，两组并发症的发生率低于文献^[13, 14]^报道。两组结节平均切除时间无统计学差异。定位时间组间比较中，四钩定位针组平均定位时间为（13.66±3.11）min，记忆合金弹簧圈组为（15.51±3.65）min，二者有统计学差异（*P*=0.001），在根据结节至胸膜不同距离分组比较时，两组定位时间均有统计学差异，而在组内比较时，记忆合金弹簧圈组当结节距离胸膜≥1.5 cm和 < 1.5 cm时，平均定位时间分别为（17.20±4.46）min和（14.91±3.15）min，二者有统计学差异（*P*=0.044），而四钩定位针组未显示有统计学差异。笔者认为该结果的出现可能是由于记忆合金弹簧圈在定位结节时，较四钩定位针相比操作稍显繁杂，穿刺针、导引针、推送针的配合使用增加了定位时间。而在定位较深位置结节时，确定定位装置释放点以及多次调整穿刺角度会延长操作时间，因而从定位时间数据分析，记忆合金弹簧圈在使用时可能更适用于距离胸膜 < 1.5 cm的肺结节。四钩定位针组出现1例中转开胸，严重的胸腔粘连导致难以继续胸腔镜探查，在开胸后顺利找到定位装置并成功实施肺结节切除。记忆合金弹簧圈组1例因切除标本未找到病灶而行妥协性扩大切除术的患者，考虑定位装置移位可能，值得注意的是，该结节到胸膜距离也超过1.5 cm。因此，四钩定位针和记忆合金弹簧圈两种定位方式均有较好的定位安全性及有效性，在定位时间方面，四钩定位针表现更佳，在使用记忆合金弹簧圈定位时，对于到胸膜距离 < 1.5 cm的肺结节效果可能更佳。不过本研究纳入样本量较小，尚需大样本研究实验来验证。

本研究在进行数据统计时未将手术总时间、术中出血量进行对比分析，考虑到手术总时间、术中出血量主要受到术式的影响，粗略地统计总手术时间^[14]^、出血量，混杂因素过多，数据可信度低。由于是回顾性分析研究，部分数据并不完善，如CT暴露次数、患者疼痛体验及术者评价、手术切除标本体积等指标未能统计，笔者观察对比中发现四钩定位针定位后部分患者疼痛体验较强，而记忆合金弹簧圈定位后情况相对较好，不过因数据不完善而未能做出统计。因定位装置原材料及制成工艺区别，记忆合金弹簧圈单价高出四钩定位针约1, 000元，但随着疼痛体验等指标的统计及使用的推广，价格方面的不足可能被其余优势所弥补。因此在两种定位方式对比时本文仍不够全面，前瞻性的研究在本中心正在积极开展。

综上，四钩定位针和记忆合金弹簧圈作为改良的定位装置，均有较好的安全性及有效性，值得临床推广和应用，四钩定位针定位操作用时更短。在使用记忆合金弹簧圈定位时，对于到胸膜距离 < 1.5 cm的肺结节效果可能更佳。
